# The effects of poverty reduction policy on health services utilization among the rural poor: a quasi-experimental study in central and western rural China

**DOI:** 10.1186/s12939-019-1099-7

**Published:** 2019-11-29

**Authors:** Qi Zou, Xiaoqun He, Zhong Li, Wanchun Xu, Liang Zhang

**Affiliations:** School of Medicine and Health Management, Tongji Medical College, Huazhong University of Science and Technology, No 13 Hangkong Road, Qiaokou District, Wuhan, 430030 Hubei China

**Keywords:** Poverty, Policy, Equality, Health services, Financial assistance, China

## Abstract

**Background:**

China poverty reduction policy (PRP) addresses two important elements: the targeted poverty reduction (TPA) project since 2015 in line with social assistance policy as national policy; and reducing inequality in health services utilization by making provision of medical financial assistance (MFA). Therefore, this study aims to assess the effects of the PRP in health services utilization (both inpatient and outpatient services) among the central and western rural poor of China.

**Methods:**

The study conducted household survey and applied propensity score matching (PSM) method to assess the effects of the PRP on health services utilization among the rural poor of Central and Western China. A sensitivity test was also performed on the PSM results to test their robustness.

**Results:**

Key findings showed 17.6% of respondents were the beneficial of PRP. The average treatment effects on the treated (ATT) of the PRP on the inpatient visits within one year was found significantly positive (*P* = 0.026).

**Conclusion:**

There has been relationship between PRP with medical financial assistance and reduction of inequality in health services utilization by the poorer, in particular to accessing the inpatient services from the county or township hospitals of China. Policy makers should pay attention for making provision of improving responsiveness of supply, when subsidizing on the demand side.

## Background

China began its reform and opening up in 1978 as the economy continued to develop rapidly. Four decades of development have enabled China’s economy to make remarkable achievements and considerably improve people’s income and living standards [1, 2], resulting in the rapid decline in the number of rural poor from 250 million in 1978 to 30 million in 2017; the corresponding proportion of people living in poverty dropped from 30.7 to 3.1% [3]. The United Nations has proposed eliminating all forms of poverty in the world by 2030, as suggested in its sustainable development goals [2]. China’s out-of-poverty population is the largest in the world [4]; thus, it has made an outstanding contribution to the achievement of the sustainable development goals [4]. However, the number of rural poor in China is still very large. There were still 30 million rural people living below the poverty line in 2017 (annual per capita disposable income of less than 2300 RMB, which was approximately one dollar per day).

Several poverty reduction policy exist in rural China to help improve the living standards and socioeconomic status (SES) of the rural poor. The TPA project and social assistance are the two most important elements of China’s PRP.

The TPA project was first proposed by President Xi Jinping in 2013 [5], and the central government made the decision to implement it in 2015 [6]. China’s TPA aims to provide targeted assistance to rural residents living below the poverty line by eliminating factors that cause poverty [5]. China’s TPA uses accurate measurement methods to identify poor households and individuals. The central and local governments help the poor emerge from poverty through strategies such as transfer payments, special industry development, employment projects and relocation for the rural poor [6]. In particular, in the field of health, the TPA has issued a special health poverty alleviation project aimed at providing rural poor people with equal access to health services through financial assistance [7].

Social assistance is another major PRP in China intended to guarantee the basic quality of life for the poor who cannot emerge from poverty through the TPA because they are unable to work, have no source of income or are elderly people and minors who are not supported by households. In 2014, the Chinese central government promulgated the Interim Measures for Social Assistance [8], which determined the current social assistance policy in the form of a national system. The minimum livelihood guarantee program (Dibao) and the poverty-stricken population support program (Wubao) are the main social assistance programs in China. Dibao is used to assist residents living below the minimum livelihood guarantee line and was first implemented in rural China in 2007 [9]. Wubao, which was first established in 2006 in the form of a government system for the rural extremely poor [10], aims to assist extremely vulnerable groups such as solitary people with no labor ability, widowed elderly people or unaccompanied children [10]. China’s social assistance program uses various strategies to meet the basic needs of the poor, such as funding some living expenditures, supporting vulnerable groups and resettling affected people. In 2017, China’s social assistance program covered approximately 45 million rural residents [3].

At present, the TPA plays a major role in poverty reduction in rural China [6]. The basic livelihood of households or individuals (especially extremely vulnerable groups) who are unable to escape poverty through industrial support or employment assistance is protected by social assistance supplements. China’s PRP can effectively increase the income of the rural poor, considerably improve their living standards and assist the beneficiaries to emerge from poverty [5, 11, 12].

Numerous reasons exist for rural Chinese residents to suffer from or return to poverty, such as disease, disasters, low education levels and unemployment. Among the different factors, disease is one of the main causes of poverty, affecting 42% of the poor population in China [13]. Low-income groups tend to be less healthy than high-income groups owing to their weak SES [14]. Because low-income groups are more prone to illness, they are more likely to utilize health services; however, compared to high-income groups, they utilize such services less often [14–19]. This phenomenon means that the income gap hinders access to health services among the poor and that extensive inequality in health services utilization exists between low-income and high-income groups.

The Chinese government launched the New Cooperative Medical Scheme (NCMS) in 2003 to reduce the health burden of rural residents and improve their access to health services. The scheme provides government cash subsidies and encourages voluntary participation among rural residents [20]. As of 2016, the NCMS covered 99.36% of all rural residents, achieving nearly full coverage [21]. The NCMS promotes registrants’ health services utilization to a certain extent and reduces their health burden [22, 23]. However, the role of the NCMS in reducing the catastrophic health expenditures of rural residents is not obvious [23–28]. Although the NCMS reduces the medical expenditures of high-income individuals, it does not effectively reduce the medical expenditures of low-income groups. Owing to this problem, the NCMS has played a limited role in reducing inequality in health services utilization [24, 25, 29].

China’s PRP provides MFA to the beneficiaries to eliminate this inequality in health services utilization and to reduce the health burden of the rural poor. The MFA supplements the weaknesses of the NCMS in reducing inequality in health services utilization [30]. The beneficiaries of the PRP receive MFA in the following aspects: 1) insurance costs for the NCMS are all paid for by the government; 2) out-of-pocket (OOP) payments for serious disease treatments are subsidized; 3) medical rehabilitation programs for the disabled are integrated into the NCMS; 4) diagnosed serious diseases are treated before payment is made; and 5) the number of serious disease insurance deductible lines is reduced, and the proportion of insurance reimbursements for serious diseases is increased [7, 8]. In 2017, 56.21 million vulnerable individuals in China received government funding for basic medical insurance costs, and 35.151 million people received direct medical assistance. The expenditures on government-funded beneficiaries with basic medical insurance are approximately 7.4 billion yuan, and the direct medical assistance expenditures are approximately 26.61 billion yuan. Thus, the beneficiaries receive direct medical assistance of 756.6 yuan per capita [3].

In short, the beneficiaries of the PRP are financially assisted by the government and society and receive MFA when they are unable to afford expenditures after treatment. From this perspective, some of the goals and important initiatives of China’s PRP to eradicate poverty include solving the problem of ‘disease-induced poverty’ among rural poor residents. The main goal of MFA is to reduce or eliminate the inequality in health services utilization caused by gaps in SES.

A previous quasi-experimental study found that TPA reduced out-of-pocket payments by 15% on average; decreased the probability of incurring catastrophic health expenditures and impoverishing health expenditures by 7.7 and 11.7%, respectively, at the household level; and within one year increased the inpatient visit rate of impoverished households by 0.035 [31]. However, the study did not indicate the impact of the PRP on the beneficiaries’ health services-seeking behavior at the individual level, and the role of the PRP in outpatient services utilization is still not clear. In addition to the above study, although other empirical studies have demonstrated that the PRP improves access to health services among the poor, unobserved variables (or hidden bias) cannot be eliminated because very few studies have applied rigorous randomized controlled trials (RCTs) or quasi-experimental methods for empirical analysis; thus, the evidence for their results and conclusions is not strong [32, 33].

## Method

### Study design

The study, which was based on a cross-section of residential health needs, aimed to evaluate the health services needs and utilization of Chinese residents. This study was conducted in two rural counties in China, namely, Dangyang and Sinan. Dangyang is a nonpoor county in Yichang City, Hubei Province, Central China, and Sinan is an impoverished county in Tongren City, Guizhou Province, Western China. A multistage stratified random sample was conducted for one-to-one interviews. Our sample for the household survey was 1355 households comprising 3983 individuals from 30 villages in Sinan and 1360 households comprising 3310 individuals from 30 villages in Dangyang.

This study used propensity score matching (PSM) to assess the effects of the PRP on the beneficiaries’ health services utilization, including outpatient and inpatient services utilization, to fill the gaps in the research methods and content in this area and provide strong evidence for how the PRP improves medical assistance.

### Data collection

We conducted a household survey in Sinan in July 2018 and in Dangyang in August 2018. Data were collected from a household-individual cross-sectional questionnaire that asked respondents about the background characteristics of their household as well as individual demographics, health status, health behaviors, health services needs and utilization and chronic disease management. We included individuals aged 15 years and older in this study. After individuals who did not match the selection criteria were excluded, our sample consisted of 6091 individuals.

### Dependent variables

As shown in Table 1, we used the characteristics of health services utilization as dependent variables. The two variables selected as indicators of the outpatient services utilization characteristics of respondents who suffered from a disease within two weeks of the survey were the two-week outpatient visit rate and the choice of outpatient institution. The two variables selected as indicators of inpatient services utilization characteristics were the inpatient visit rate within one year and the choice of inpatient institution. We defined the two-week outpatient visit rate as the ratio of the number of cases with outpatient visit to the number of cases with any disease within two weeks. We defined the inpatient visit rate within one year as the ratio of the number of inpatient who incurred at least one hospitalization to the total number of respondents within one year.

**Table 1:** Health services utilization and background characteristics

Variables, n (%) or mean (SD)	Total (*n* = 6091)	Beneficiaries (*n* = 1069)	Non-beneficiaries (*n* = 5022)	*P*-value
Dependent variables outpatient services utilization within two weeks				
Any outpatient visit				0.054
Yes	461 (18.84)	96 (22.12)	365 (18.13)	
No	1986 (81.16)	338 (77.88)	1648 (81.87)	
Outpatient institutions				0.310
Village clinic	214 (44.12)	35 (33.65)	179 (46.98)	
Town health center	99 (20.41)	27 (25.96)	72 (18.90)	
Public hospital above county-level	142 (29.28)	38 (36.54)	104 (27.30)	
Private hospital or clinic	30 (6.19)	4 (3.85)	26 (6.82)	
Inpatient services utilization of patients within one year				
Any inpatient visit				< 0.001
Yes	1188 (19.50)	276 (25.82)	912 (18.16)	
No	4903 (80.50)	793 (74.18)	4110 (81.84)	
Inpatient institutions				0.274
Town health center	351 (27.04)	83 (27.76)	268 (26.83)	
Public hospital of county-level	742 (57.16)	179 (59.87)	563 (56.36)	
Public hospital above city-level	164 (12.63)	28 (9.36)	136 (13.61)	
Private hospital or other institutions	41 (3.16)	9 (3.01)	32 (3.20)	
Control variables				
Area				< 0.001
Sinan County	3090 (50.73)	754 (70.53)	2336 (46.52)	
Dangyang County	3001 (49.27)	315 (29.47)	2686 (53.48)	
Distance between residence and nearest health services institutions (km)				< 0.001
≤ 1	3037 (49.90)	503(47.14)	2534(50.49)	
1~2	1913 (31.43)	292(27.37)	1621(32.30)	
2~3	743 (12.21)	182(17.06)	561(11.18)	
3~4	158 (2.60)	25(2.34)	133(2.65)	
> 4	235 (3.86)	65(6.09)	170(3.39)	
Household per capita annual income level				< 0.001
Low income group	1218 (20.00)	368 (34.42)	850 (16.93)	
Lower income group	1219 (20.01)	243 (22.73)	976 (19.43)	
Middle income group	1210 (19.87)	193 (18.05)	1017 (20.25)	
Higher income group	1226 (20.13)	152 (14.22)	1074 (21.39)	
Highest income group	1218 (20.00)	113 (10.57)	1105 (22.00)	
Gender				0.424
Male	2992 (49.17)	537 (50.28)	2455 (48.93)	
Female	3093 (50.83)	531 (49.72)	2562 (51.07)	
Age group				< 0.001
15~24	489 (8.03)	107 (10.01)	382 (7.61)	
25~44	1070 (17.57)	164 (15.34)	906 (18.04)	
45~64	2856 (46.89)	454 (42.47)	2402 (47.83)	
≥ 65	1676 (27.52)	344 (32.18)	1332 (26.52)	
Marital status				< 0.001
Unmarried	601 (9.91)	156(14.70)	445(8.89)	
Married	4851 (79.98)	768(72.38)	4083(81.59)	
Divorced	54 (0.89)	15(1.41)	39(0.78)	
Widowed	548 (9.04)	116(10.93)	432(8.63)	
Other	11 (0.18)	6(0.57)	5(0.10)	
Education				< 0.001
Illiteracy	1062 (17.48)	253(23.73)	809(16.14)	
Elementary school or junior high school	4038 (66.45)	691(64.82)	3347(66.79)	
High school or secondary vocational and technical school	803 (13.21)	90(8.44)	713(14.23)	
University degree	174 (2.86)	32(3.00)	142(2.83)	
Severity of disease within two weeks				< 0.001
Not at all serious	30 (1.22)	4 (0.92)	26 (1.29)	
Not too serious	386 (15.74)	47 (10.83)	339 (16.84)	
Generally serious	934 (38.08)	125 (28.80)	809 (40.19)	
Relatively serious	989 (40.32)	223 (51.38)	760 (37.75)	
Very serious	114 (4.65)	35 (8.06)	79 (3.92)	
The utility index of HRQoL	0.854 (0.002)	0.793 (0.006)	0.866 (0.002)	< 0.001

Note: In the statistical analysis of dependent variables, for the variable ‘any inpatient visit’, we used an individual as an observation case. For other dependent variables, we used an outpatient or inpatient record as an observation case

### Control variables

The control variables used to assess the propensity scores are shown in Table 1. The household-level variables included distance between residence and the nearest health services institution and annual household income per capita. The variables at the individual level included gender, age, marital status, educational level and the utility index of the health-related quality of life (HRQoL). Moreover, we included clinical characteristic-related variables to the matching models to improve their accuracy in terms of choices of health services institutions. The types of diseases were classified as clinical characteristics related to the control variables. Among the control variables, we classified the respondents into 5 groups based on their annual household income per capita, namely, low-income group, lower-income group, middle-income group, higher-income group and highest-income group. We used the EQ-5D-3 L version of the 5th China National Health services Survey to measure the utility index of the HRQoL, which was applied to those who were 15 years old and older [34]. The EQ-5D-3 L method was used to calculate the time trade-off value to calculate the utility index of the HRQoL, and the evaluation method was revised by Professor Liu et al. according to the health status of Chinese residents, decreasing the gap between the utility index and their HRQoL [35]. We used the ICD-10, which was proposed by the WHO, to classify the disease types of the two-week outpatients and one-year inpatients [36]. The distribution of disease types among the beneficiaries and nonbeneficiaries is shown in Table 5 of the Appendix.

### Statistical analysis

We included the household- and individual-level information of all of the respondents in the data analysis. We used the likelihood chi-square test for the categorical variables and two independent sample t-tests or nonparametric tests for the continuous variables to compare the differences in health services utilization and background characteristics between the beneficiaries and nonbeneficiaries. Then, PSM was used to assess the ATT of the PRP on rural residents’ health services utilization. First, we used the propensity score to find cases of nonbeneficiaries that are as similar as possible to those of beneficiaries. These cases constituted the ‘control group’, whereas the sample of beneficiaries included in the matching models was the ‘treatment group’. The pretreatment characteristics of the treatment group and the control group were as similar to one another as possible. The variation in the control variables was derived from the treatment effects by controlling the pretreatment characteristics. We assessed the propensity scores by employing logistic regression [37, 38].

We chose the one-to-one neighbor matching method with replacements to select the treatment group and control group cases. We selected the two cases in the treatment and control groups with the closest match of propensity scores. The 95% confidence intervals were estimated by a bootstrap method. We used a balance test to estimate the bias before and after matching. Subsequently, we calculated the ATT of the PRP on beneficiaries’ health services utilization and its significance in evaluating policy effectiveness. Finally, we used the Rosenbaum method to conduct a sensitivity analysis to determine the existence of unobserved variables that can affect rural residents’ access to PRP coverage and health services utilization [39].

We used the Epidata software version 3.1 to perform double entry on the questionnaire data and to verify the entry results. The data were initially processed by MS Excel 2016 and analyzed by Stata software v.12 SE.

## Results

### Health services utilization and background characteristics

Table 1 shows the health services utilization and background characteristics of the beneficiaries and nonbeneficiaries of the PRP. Overall, the proportion of Chinese rural residents receiving PRP benefits was 17.55% (1069/6091), the two-week outpatient visit rate was 18.84% (461/2447), and the inpatient visit rate within one year was 19.50% (1188/6091).

Specifically, the two-week outpatient visit rate (22.12%) and inpatient visit rate within one year (25.82%) were higher for the beneficiaries than for the nonbeneficiaries (18.13 and 18.16%). However, there was no significant difference in the choices of health services institutions between the two groups. Of the beneficiaries, 87.63% chose public hospitals at the county or township level to obtain inpatient services, and 59.61% chose village clinics or town health centers to obtain outpatient services. In terms of background characteristics, 6.09% of the beneficiaries were more than 4 km from the nearest health services institutions, compared to 3.39% of the nonbeneficiaries. The per capita annual income of 57.16% of the beneficiaries was below the middle income level, compared to 36.36% of the nonbeneficiaries. Of the beneficiaries, 32.18% were aged 65 and older, compared to 26.52% of the nonbeneficiaries. Regarding marital status, 72.38% of the beneficiaries were married, compared to 81.59% of the nonbeneficiaries. Regarding educational status, 23.73% of the beneficiaries were illiterate, compared to 16.14% of the nonbeneficiaries. Of the beneficiaries who were sick within two weeks, 8.06% felt that their disease was very serious, compared to 3.92% of the nonbeneficiaries (3.92%). The average utility index of the HRQoL for the beneficiaries was 0.793 ± 0.006, compared to 0.866 ± 0.002 for the nonbeneficiaries.

### The quality of the PSM models

Table 2 shows the quality of the matching models before and after the best matching sample. We determined the final analytical model based on the matching quality, which was evaluated based on the resulting quality parameters, including the mean and median absolute bias of variables, the pseudo-R^2^ and the standard likelihood ratio test χ^2^. The results showed that the mean and median bias of the treatment and control variable sets of all the models were significantly reduced. Most of the observed variables in the two groups had a mean bias that was less than or close to 5%. The pseudo-R^2^ was derived from the regression of the propensity scores of the variables of the matched and unmatched samples. If the quality of the propensity score match was good, then the matched pseudo-R^2^ was significantly reduced [37, 38]. The pseudo-R^2^ was significantly reduced in all of our PSM models after they were matched. The variables’ likelihood-ratio test of joint nonsignificance was nonsignificant, indicating that the distribution of all the variables in the two groups was similar after matching.

**Table 2:** The quality of the PSM models

Matching model	Sample	Mean Bias	Median bias	Pseudo-R^2^	LR χ^2^	*P* > χ^2^
Any outpatient visit	Unmatched	22.2	15.8	0.125	276.16	0.000
	Matched	3.9	3.1	0.005	5.97	0.918
Outpatient institutions	Unmatched	24.4	17.6	0.173	68.82	0.000
	Matched	7.9	7.6	0.027	7.24	0.841
	Matched	10.2	7.5	0.036	9.29	0.751
Any inpatient visit	Unmatched	21.1	17.7	0.092	506.15	0.000
	Matched	3.0	2.7	0.002	5.46	0.859
Inpatient institutions	Unmatched	17.2	13.4	0.073	96.95	0.000
	Matched	3.0	3.4	0.003	2.01	0.996
	Matched	5.2	4.4	0.011	7.94	0.719

### The ATT of the PRP on health services utilization

#### The ATT of the PRP on outpatient services utilization

Table 3 shows the ATT of the PRP on health services utilization. There were no significant effects of the PRP on the two-week outpatient visit rate for rural residents (*P* = 0.976). Similarly, the PRP had no significant effects on rural residents’ choices of the level of outpatient institutions (*P* = 0.945).

**Table 3:** The ATT of the PRP on health services utilization

Outcomes	Means after matching		Average treatment effects			Number of cases on support
	Treatment	Control	ATT	SE	*P*-value	
Any outpatient visit	0.2206	0.2218	− 0.0012	0.0327	0.976	2352
Outpatient institutions	2.092	2.107	−0.015	0.161	0.945	466
Any inpatient visit	0.2599	0.2084	0.0515	0.0192	0.026	5932
Inpatient institutions	1.857	1.853	0.004	0.070	0.961	1183

#### The ATT of the PRP on inpatient services utilization

During 2017, the ATT of the PRP for the inpatient visit rate within one year was 0.0515 and significant (*p* < 0.05). The results showed that the PRP had increased the inpatient visit rate within one year by approximately 5 percentage points. Meanwhile, the ATT of the PRP on rural residents’ choices of outpatient institutions (*P* = 0.961).

### Sensitivity analysis for hidden bias

The ATT obtained using PSM was based on the assumption that no influence of unobserved variables existed. However, a hidden bias may occur, and the matching results may no longer be robust if unobserved variables exist that can affect the access of the poor to PRP coverage and health services utilization [39]. Here, we used the sensitivity parameter gamma coefficient (Γ), which was proposed by Rosenbaum, to measure the existence of a hidden bias in the model. If the background characteristics of the treatment and control groups were consistent, then Γ was calculated based on the ratio of the treatment effects of the unobserved variables to the treatment group sample and the treatment effects of the control sample [39].

The basic principle of the Rosenbaum method is to use Γ to measure the effects of the hidden bias on the processing estimates. If Γ = 1, then the matching model has no hidden bias, and the outcomes are very robust. As Γ increases, the level of the hidden bias increases simultaneously. Given that the unobserved variables cannot be measured, the Γ value can likewise not be directly calculated; thus, the Γ value is determined based on its value before the significance of the estimated treatment effects changes. The assumed maximum Γ value is called the ‘Rosenbaum boundary’. In the current study, the criteria for the Γ value were not defined; however, in the social sciences, the Γ value is usually limited between 1 and 2 to indicate that a hidden bias does not exist [40].

Table 4 shows the results of the sensitivity analysis of the matching models. For all of the matching models, Γ = 1, showing that the processing effects were not sensitive to hidden bias, and no unobserved variables existed that would affect the access of the poor to the PRP.

**Table 4:** Sensitivity analysis for matching models

Γ	*P*-value			
	Any outpatient visit	Outpatient institutions	Any inpatient visit	Inpatient institutions
1	0.455	0.433	0.101	0.639
1.5	0.991	0.938	0.998	0.998
2	1.000	0.997	1.000	1.000
2.5	1.000	1.000	1.000	1.000
3	1.000	1.000	1.000	1.000

## Discussion

We used a quasi-experimental method to assess the effects of China’s PRP on health services utilization and obtained several important findings. Our main finding revealed that the PRP has made some achievements in reducing inequality in health services utilization among the poor. Within one year of the survey, under the premise of consistent health services demand, the beneficiaries occurred more inpatient services utilization. However, no significant difference existed in outpatient services utilization between the treatment group and the control group. The inpatient visit rate within one year was approximately 5% higher for the treatment group than for the control group. These findings showed that the PRP mainly protects the poor with serious diseases from catastrophic health expenditures.

Most rural residents in China experience inequality in health services utilization owing to the income gap [19, 41]. Our study found that the PRP has alleviated this situation to a certain extent and has mainly improved the beneficiaries’ access to inpatient services. It is worth noting that the beneficiaries of the PRP did not appear to make higher-level choices of health services institutions for inpatients, as 87.63% of the inpatients in the treatment group chose public hospitals at the county or township level. However, only 59.61% of the outpatients chose primary health services institutions (village clinics or town health centers) for treating diseases that occurred within two weeks of the survey, which is a large gap from China’s goal of guiding patients to seek health services at primary health services institutions as well as one of the main goals of establishing a hierarchical diagnosis and treatment system in China [42, 43]. The hierarchical diagnosis and treatment system aims to promote the rational allocation of medical resources and promote equal access to basic health services by dividing health services institutions geographically into three different tiers in rural China: county hospitals, town health centers and village clinics [42, 44]. Four principles are suggested by the system to optimize health services provision: the initial diagnosis and treatment should be made by primary health services institutions; a bidirectional patient referral should be executed to provide optimal health services; acute and chronic diseases should be treated distinctively by the appropriate channels in health services institutions at different tiers; and medical resource optimization should be carried out within the three tiers rather than within an individual health services institution [42, 44].

The explanation of the supply side for the above results is that the main goal of the PRP in the health field is to guarantee basic health services and the treatment of serious diseases but not to provide excessive health services [7, 8]; thus, the PRP does not encourage the beneficiaries to acquire a high level of health services to avoid moral hazard. Outpatient expenditures are usually small and unlikely to cause catastrophic health expenditures among the poor; thus, the PRP is not concerned about outpatient assistance.

On the demand side, Yan-Ning Li et al.’s survey in rural areas of Guangxi Zhuang Autonomous Region (Southwestern Province) showed that the most important factors affecting residents’ two-week illness rate were need factors (such as illness) rather than economic factors [45]. After using PSM to eliminate the effects of disease characteristics and the HRQoL on residents’ health services utilization, we also found that the PRP had no effects on residents’ two-week outpatient visit rates. In addition, Lu Zhang et al.’s study in rural areas of Gansu Province (Northwestern Province) showed that increasing outpatient reimbursement rates had no effect on the choice of outpatient institution for type-2 diabetes patients [46]. Only 28% of patients with type-2 diabetes chose village clinics, and 33% chose town health centers for health services [46]. The main reason is that Chinese residents’ distrust in primary health services institutions was higher than their distrust in hospitals at the county level and above [47], and this high distrust of primary health institutions was strongly associated with increased hospital outpatient services utilization [47]. Some studies have shown that convenience is also an important factor that affects outpatient services utilization among Chinese residents [45, 48, 49]. In general, the impact of the PRP on outpatient services utilization among rural residents is minimal. Instead, residents’ disease characteristics, health status, trust in health services institutions, and convenience are the main factors affecting outpatient services utilization.

Different from outpatient services utilization, many studies have shown that economic factors and need factors both play dominant roles in inpatient services utilization among Chinese residents [45, 48, 50]. Meanwhile, a Chinese nationally representative survey from 1993 to 2008 showed that with the same needs for health services, rich rural residents utilized more health services than poor rural residents, and inpatient services utilization was more inequitable [51]. Our study shows that after controlling for demographic socioeconomic characteristics and need factors (HRQoL), the PRP increased the inpatient visit rate of the beneficiaries by approximately 5 percentage points, indicating that MFA provided by the PRP greatly reduced the inequality of inpatient services utilization caused by the income gap.

In general, the PRP has achieved some goals to improve health services utilization for the beneficiaries, mainly by increasing their inpatient visit rate. Compared to the nonbeneficiaries, the beneficiaries were more willing and able to obtain inpatient services when they suffered from serious diseases because they were safeguarded by the PRP [18]. This finding indicates that the PRP has made progress in reducing inequality in the health services utilization of the rural poor, including the formulation and improvement of policies, the targeted identification of the poor and the increase and management of MFA funds [52]. Despite these achievements, further PRP improvements are needed.

The MFA strategies of the PRP have achieved good results in reducing the inequality of inpatient services utilization among the rural poor in China, indicating that these strategies should continue to be implemented. However, the PRP has not been able to improve the equality of the outpatient services utilization of the rural poor on the demand side, and government departments should improve the responsiveness of the supply side to meet the outpatient services needs of the rural poor. Specifically, the government should guide primary health services institutions to make greater efforts to improve the satisfaction of rural residents, with the aim of encouraging the rural poor to initially access diagnosis and treatment in primary health services institutions when needed. Primary health services institutions in rural China generally have weak services capacity and poor medical quality, which is the main reason for the low satisfaction of rural residents in China [53, 54]. Therefore, improving the services capacity and medical quality of primary health services institutions will guide patients to choose primary health services institutions, which should be the core task of China’s rural health policies [53–55]. The deployment and training of health technicians and general practitioners is the main measure to achieve this goal [53, 55, 56], [57]. Through these efforts, the efficiency of primary health services institutions and the health outcomes of the rural poor will improve, and the financial risks of the rural poor due to catastrophic health expenditures will decrease.

## Limitations

A few limitations exist in our study. First, we use cross-sectional data to conduct the study, which cannot longitudinally observe the changes in health services utilization among the beneficiaries of the PRP in the early, middle and late stages of the benefits. Second, although we use quasi-experimental methods for the analysis, given the limited number of variables and the sample size, the matching models still contain bias, which reduces the accuracy of the results. In particular, due to the limitations of the survey, factors such as social culture, health literacy, and psychology are not included in the control variables, and these factors may have different degrees of effects on health services utilization among the rural poor. These limitations should be further addressed in future research.

## Conclusions

Our study found that the PRP has to some extent reduced inequality in health services utilization among rural residents, which is caused by the income gap, mainly by increasing the beneficiaries’ inpatient visit rate within one year. In addition, the vast majority of the beneficiaries choose inpatient institutions at the county or township level. These results show that the PRP plays a positive role in the beneficiaries’ inpatient services utilization. However, the PRP had no effects on the two-week outpatient visit rate and choice of outpatient institution among the poor. Moreover, due to weak services capacity and poor medical quality, a considerable number of the beneficiaries failed to initially access health services at primary health services institutions after they became ill. The limited role of the PRP in outpatient services leads to disordered treatment and insufficient reduction of medical burden among the poor. Policy makers need to pay attention to improving the responsiveness of the primary health services system and guiding patients to initially access health services in primary health services institutions by improving the services capacity and medical quality of primary health services institutions.

## Data Availability

The datasets used and analyzed during the current study are available from the corresponding author on reasonable request.

## Appendix

**Table 5:** The distribution of diseases classification for beneficiaries and non-beneficiaries

Diseases Classification	Number of outpatients			Number of inpatients		
	Beneficiaries	Non-beneficiaries	Total	Beneficiaries	Non-beneficiaries	Total
Certain infectious and parasitic diseases	9	14	23	5	9	14
Neoplasms	17	28	45	11	39	50
Diseases of the blood and blood-forming organs and certain disorders involving the immune mechanism	3	4	7	3	4	7
Endocrine, nutritional and metabolic diseases	26	182	208	11	37	48
Mental and behavioural disorders	13	10	23	7	10	17
Diseases of the nervous system	13	40	53	8	21	29
Diseases of the eye and adnexa	8	25	33	6	28	34
Diseases of the ear and mastoid process	2	10	12	2	5	7
Diseases of the circulatory system	82	597	679	52	187	239
Diseases of the respiratory system	64	245	309	49	146	195
Diseases of the digestive system	29	139	168	24	95	119
Diseases of the skin and subcutaneous tissue	2	18	20	2	5	7
Diseases of the musculoskeletal system and connective tissue	80	409	489	36	97	133
Diseases of the genitourinary system	27	98	125	19	93	112
Pregnancy, childbirth and the puerperium	0	1	1	4	39	43
Certain conditions originating in the perinatal period	0	1	1	0	1	1
Congenital malformations, deformations and chromosomal abnormalities	3	3	6	0	1	1
Symptoms, signs and abnormal clinical and laboratory findings, not elsewhere classified	25	85	110	23	30	53
Injury, poisoning and certain other consequences of external causes	27	95	122	30	125	155

Note:The diseases classification is based on the ICD-10 released by the WHO

## Abbreviations

ATT: Average treatment effects on the treated
Dibao: Minimum livelihood guarantee program
HRQoL: Health-related quality of life
NCMS: New Cooperative Medical Scheme
OOP: Out-of-pocket
PRP: Poverty reduction policy
PSM: Propensity score matching
SES: Socioeconomic status
TPA: Targeted poverty alleviation
Wubao: Poverty-stricken population support program
